# Inducing Cytotoxicity in Colon Cancer Cells and Suppressing Cancer Stem Cells by Dolasetron and Ketoprofen through Inhibition of RNA Binding Protein PUM1

**DOI:** 10.3390/toxics11080669

**Published:** 2023-08-03

**Authors:** Ravi Gor, Ali Gharib, Priya Dharshini Balaji, Thirumurthy Madhavan, Satish Ramalingam

**Affiliations:** 1 Department of Genetic Engineering, School of Bio-Engineering, SRM Institute of Science and Technology, Kattankulathur, Chengalpattu 603203, Tamil Nadu, India; ravigor95@gmail.com (R.G.); ag8855@srmist.edu.in (A.G.); 2 Computational Biology Laboratory, Department of Genetic Engineering, School of Bio-Engineering, SRM Institute of Science and Technology, Kattankulathur, Chengalpattu 603203, Tamil Nadu, India; pb5966@srmist.edu.in (P.D.B.);

**Keywords:** post-transcriptional gene regulation, Pumilio1, cancer stem cells, toxicity, dolasetron, ketoprofen

## Abstract

Clinical trials of new drugs often face a high failure rate of approximately 45 percent due to safety and toxicity concerns. Repurposing drugs with well-established safety profiles becomes crucial in addressing this challenge. Colon cancer ranks as the third most prevalent cancer and the second leading cause of cancer related mortality worldwide. This study focuses on the RNA-binding protein pumilio1 (PUM1), a member of the PUF family involved in post-transcriptional gene expression regulation. By utilizing molecular docking techniques and FDA-approved drugs, potential inhibitors against PUM1 were identified. Notably, dolasetron and ketoprofen demonstrated promising results, exhibiting strong binding affinity, hydrophobic interactions, and favorable chemical reactivity according to Conceptual-DFT calculations. Both compounds effectively reduced cell viability, with IC50 values of 150 µM and 175 µM, respectively and shows long term inhibitory effects as seen by reduced in number of colonies. Moreover, they exhibited inhibitory effects on colon cancer stem cells, as indicated by reduced colonospheroid size and numbers. Apoptosis is induced by these compounds and has triggered activation of executioner caspase 3/7 in HCT116 cells which is evident through a caspase 3/7 assay and AO/EB staining, while the non-toxic effect of these compounds was evident from viability against non-cancerous cell line and hemolysis assay. Additionally, the treatment group showed a significant decrease in PUM1 and cancer stem cell markers expression compared to the control group. In conclusion, this study highlights the potential of targeting PUM1 as a novel approach to colon cancer treatment. Dolasetron and ketoprofen demonstrate promise as effective anti-cancer and anti-cancer stem cell drugs, inducing apoptosis in colon cancer cells through inhibition of PUM1.

## 1. Introduction

Clinical trials experience a failure rate of approximately 45 percent due to concerns related to the safety and toxicity of drugs under investigation. However, repurposing drug candidates offers the advantage of having well-established safety and toxicity profiles. This approach can significantly reduce the average drug development time by 5–7 years, benefiting both patients and drug developers [1]. Among various cancer types, colon cancer ranks as one of the most prevalent tumors worldwide, alongside lung, prostate, and breast cancer, and it stands as the third most common type of cancer. While traditionally more prevalent in developed countries, the incidence of colon cancer is now rising in developing nations due to dietary changes and the adoption of Western culture. Particularly, significant increase in the prevalence of colon cancer have been observed in Asia and Eastern European countries, while age-adjusted rates have remained relatively stable in Western Europe and Oceania. Early treatment of colon cancer leads to a more favorable prognosis, and several new target-oriented drugs have exhibited significant activity and efficacy [2].

Pumilio1 (PUM1), a member of the Pumilio and fem3 binding protein family (PUF), is an evolutionarily conserved RNA-binding protein (RBP) that plays a crucial role in gene regulation across various organisms. PUM1 consists of eight copies of 36 amino acid repeats called PUF repeats, with repeat 7 (R7) being highly conserved among different species [3,4]. Targeting the PUM1 protein and its motifs could disrupt interaction with target mRNAs, leading to the dysregulation of cellular functions. Notably, PUM1 has been implicated in the progression of various cancers, including non-small cell lung, ovarian, prostate, and colon cancer. Increased expression of PUM1 has been directly correlated with cancer progression, while its silencing has demonstrated reduced cell proliferation, migration, and invasion ability [5]. In colon cancer, higher PUM1 expression has been observed in tumor tissues and cell lines, and overexpression of PUM1 significantly promoted cell proliferation, colony formation, cell migration, and colonosphere formation [6]. Activation of the PERK/eIF2/ATF4 signaling pathway has also been implicated in PUM1-mediated tumor progression in pancreatic adenocarcinoma cells [7].

Recent studies have highlighted the potential of PUM1 as a therapeutic target for cancer treatment. For instance, targeting PUM1 using siRNA-encapsulated nanoparticles reduced colorectal tumor growth in a murine orthotopic colon cancer model [8]. Additionally, the plant flavonoid morin has been found to inhibit colon cancer stem cells in vitro by suppressing PUM1 expression [9]. Such findings suggest that PUM1 represents a promising target for reducing cancer growth and underscores its potential as a novel therapeutic target for colon cancer and other cancer types.

To identify potential inhibitors for the PUM1 protein, a drug repurposing approach is being employed. Drug repurposing involves the identification of new targets and alternative therapeutic uses for approved drugs [10]. Molecular docking, a structural-based computational strategy, is employed to predict the binding interactions between FDA-approved drugs (ligands) and the therapeutic target PUM1. This approach aids in selecting the top compounds with the highest binding affinity. In this study, molecular docking was performed with FDA-approved compounds in the proposed binding site (R7) of PUM1. The ten compounds with the highest binding affinity scores were further analyzed using conceptual density functional theory (c-DFT) to understand the stability and interactions between the protein-ligand complexes. Molecular dynamic simulation (MDS) was performed to study the dynamic interaction between the top two compounds, dolasetron and ketoprofen, with the PUM1 target protein. The molecular dynamic trajectory was analyzed using in-built gromacs packages. In vitro assays, including MTT and colonospheroid assays, were conducted to evaluate the anti-cancer and anti-cancer stem cell potential of the selected compounds. Apoptosis potential was assessed using dual acridine orange/ethidium bromide (AO/EB) staining and an Apo-one Homogeneous Caspase-3/7 assay. Furthermore, immunofluorescence was performed with PUM1 and some colon cancer stems markers such as DCLK1 and CD133. In this research, we have identified new target for FDA-approved drugs and validated the same using in silico and in vitro experiments.

## 2. Materials and Methods

### 2.1. Preparation of Protein for Molecular Docking and Molecular Dynamic Simulation

**Figure 1:**
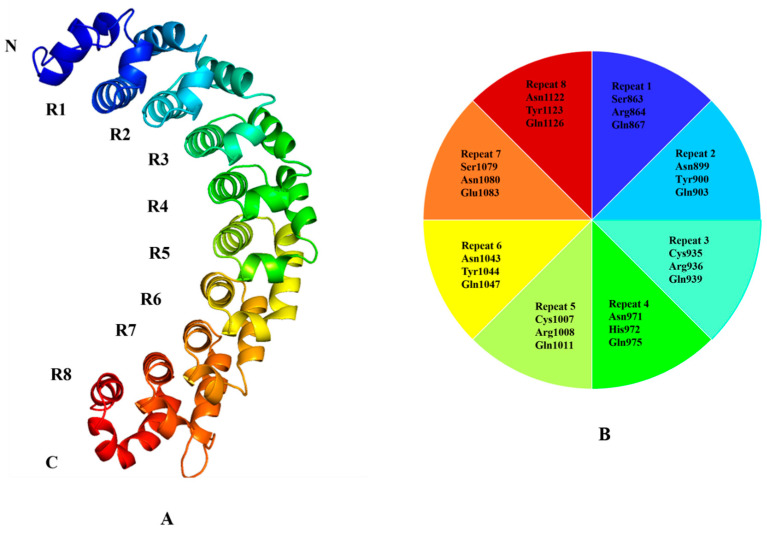
(**A**) Crystal structure of PUM1 protein (PDB ID: 1M8Z) The helical repeats are colored (spectrum) and labeled from R1 to R8, along with the N and C terminal of protein. (**B**) The active site residues of the PUM1 protein that make contact with the RNA base from R1 to R8 are depicted.

The PUM1 protein (PDB ID: 1M8Z) was obtained from the Protein Data Bank (http://www.rcsb.org/) and visualized using PyMOL (http://www.pymol.org/) as shown in Figure 1. The crystal structure of the protein contained co-crystallized water molecules and co-factors and they were removed for docking purposes. The protein was prepared using a molecular docking algorithm, which involved adding polar hydrogen atoms and charges using the Kollman method and computing Gasteiger charges with default parameters [11,12].

### 2.2. Preparation of Ligand for Molecular Docking and Molecular Dynamic Simulation

FDA-approved drugs were downloaded from the ZINC-15 database. The drugs, in SDF file format, were converted to PDBQT format using the OpenBabel version 3.1.1 software for molecular docking calculations. The drugs were optimized using the MMFF94 force field and subsequently used for molecular docking studies [11].

### 2.3. Active Site Prediction for Molecular Docking and Molecular Dynamic Simulation

The RNA-binding protein PUM1 has been reported to have eight binding sites [3]. RNA binds to the concave surface of the PUM1 protein, and each of the eight protein repeats contacts a different RNA base through three amino acid side chains. The active site residues of PUM1 include Ser863, Arg864, Gln867, Asn899, Tyr900, Gln903, Cys935, Arg936, Gln939, Asn971, His972, Gln975, Cys1007, Arg1008, Gln1011, Asn1043, Tyr1044, Gln1047, Ser1079, Asn1080, Glu1083, Asn1122, Tyr1123, and Gln1126 as shown in Figure 1.

### 2.4. Molecular Docking

Molecular docking is a computational procedure used to evaluate the binding affinities and interactions of small molecules. In this study, docking was performed using AutoDock Vina, an interactive molecular graphics tool, to compute and display the potential docking modes for protein–ligand pairings ranked by their binding affinities [13]. The binding conformation of the ligand inside the PUM1 protein was defined by a grid box that allowed the ligand atoms to explore different conformations. The docking results were ranked based on a scoring algorithm [11].

### 2.5. Conceptual Density Functional Theory (C-DFT) Analysis

Conceptual Density Functional Theory (C-DFT) is a quantum-mechanical atomistic simulation method used to calculate various attributes of atomic systems [14]. The method employs ten global reactivity descriptors and their derivatives as molecular descriptors. These descriptors include total energy (E; in eV), molecular dipole moment (Dp; in Debye units), Highest Occupied Molecular Orbital (HOMO) (EHOMO; in eV), Lowest Unoccupied Molecular Orbital (LUMO) (ELUMO; in eV), HOMO-LUMO gap (ΔE; in eV), absolute hardness (η; in eV), electronegativity (χ), global softness (σ; in eV−1), chemical potential (μ; in eV), and electrophilicity index (ψ; in eV−1). The selected ligands were optimized using the Becke-3-parameter, Lee–Yang–Parr (B3LYP) function with the 311G (2d, p) basis set in Gaussian-16 software (http://gaussian.com/gaussian16/, accessed on 18 June 2023) to determine these descriptors [15,16,17].

### 2.6. Molecular Dynamics Simulation

Molecular dynamics (MD) simulations were performed to investigate the stable binding mode of dolasetron and ketoprofen. GROMACS version 5.0 software was used to carry out a 100 ns MD simulation, allowing exploration of the ligand’s molecular interactions and stability. The coordinates for the MD simulation were obtained using the PRODRG server. The GROMOS96 54a7 force field was applied to the system, and the SPC water model was used as the solvent. The system was equilibrated for potential energy, temperature, and pressure until they reached stability. Structural and conformational changes during the simulation were investigated using Root Mean Square Deviation (RMSD), Root Mean Square Fluctuation (RMSF), and Hydrogen Bond (H-bond) analysis [18].

### 2.7. Cell Lines and Cell Culture

Human colorectal carcinoma cells (HCT116) and human embryonic kidney 293 cells (HEK293) were obtained from the NCCS, Pune. Cell culture reagents were purchased from Sigma Aldrich. The cells were cultured in Dulbecco’s Modified Eagle Medium (DMEM) containing 10% (*v*/*v*) fetal bovine serum (FBS), 1% antibiotic, and antimycotic solution. All cells were maintained at 37 °C in a humidified atmosphere with 5% CO_2_.

### 2.8. MTT Cell Viability Assay

The MTT (3-(4,5-dimethylthiazol-2-yl)-2,5 diphenyl tetrazolium bromide) was used to assess cell viability [19,20]. HCT116 human colon cancer cells and HEK293 human embryonic kidney cells were seeded at a density of 5000 cells per well in a 96-well plate. The cells were treated with different concentrations (50, 100, 150, and 200 µM) of dolasetron and ketoprofen and incubated for 48 h. After the incubation period, DMSO was added to solubilize the formazan crystals, and the absorbance was measured at 570 nm using a multi-plate reader. The IC50 value of the drug was determined by plotting the graph of drug concentration versus the percentage viability relative to the control. Further, the selectivity index was measured with the percentage viability at the IC50 value using below mentioned formula,

$$Selectivity\;Index=\frac{Percentage\;viability\;of\;non-cancerous\;cells\;\left(HEK293\right)}{Percentage\;viability\;of\;cancerous\;cells\;(HCT116)}$$

### 2.9. Hemolysis Assay

Assessing the rate of hemolysis is a commonly used method to determine the cytotoxic effects of various substances [21]. Briefly, fresh human blood was collected and stabilized using EDTA as an anticoagulant. A total of 2 mL of whole blood sample was mixed with 4 mL of phosphate-buffered saline (PBS) and subsequently subjected to centrifugation at 10,000× *g* for 5 min to isolate red blood cells (RBCs). The RBCs were then washed five times with 10 mL of PBS and ultimately diluted to a volume of 20 mL using PBS. For the experimental group, 0.1 mL of the diluted RBC suspension was exposed to IC50 concentrations of dolasetron and ketoprofen, while the positive control group was exposed to distilled water and the negative control group was exposed to PBS. Each group consisted of three tubes, which were then incubated at 37 °C for 24 and 48 h in a dedicated incubator. Upon completion of the incubation period, the samples were centrifuged again at 10,000× *g* for 5 min. A volume of 100 µL of the supernatant from each sample was transferred to a 96-well plate, and the absorbance was measured using a microplate reader at a wavelength of 590 nm. The extent of hemolysis was quantified using the hemolytic ratio, which was calculated using the following formula:

$$Hemolysis\;ratiio=\frac{OD\left(test\right)-OD\;(negative\;control)}{OD\left(positive\;control\right)-OD\;(negative\;control)}\times 100$$

This formula allowed for the determination of the hemolytic degree in relation to the controls [22,23].

### 2.10. Colony Formation Assay

In a 6-well plate, 500 cells were seeded and incubated overnight at 37 °C. The next day, the medium was replaced with an IC50 value of dolasetron-containing medium, ketoprofen-containing medium, or regular medium (control). After 48 h of incubation, the drug-containing medium was replaced with a fresh medium, and the cells were further incubated for ten days. The medium was then removed, and the cells were washed with PBS before being fixed with 10% formalin at room temperature. After fixation, the cells were washed twice with PBS and stained with 1% crystal violet in 10% ethanol. Colonies were counted in treated wells and compared to the untreated control [9,24].

### 2.11. Spheroid Formation Assay

The Spheroid Assay was conducted in 6-well poly-HEMA (P3932) coated tissue culture plates. A total 1000 cells were seeded in each well, and the IC50 values of dolasetron and ketoprofen were used for treatment in separate wells, while untreated wells served as controls. The plate was incubated for seven days in a humidified atmosphere with 5% CO_2_ to allow spheroid formation. The spheroid medium included DMEM, EGF, and B-27 as supplements. The total number of spheroids formed after incubation were counted and represented as percentage compared to the control wells [9,24].

### 2.12. Dual Acridine Orange (AO)/Ethidium Bromide (EB) Fluorescence Staining and Caspase-3/7 Activity Assay

HCT116 colon cancer cells were seeded in 24-well plates with a density of 1 × 10^5^ cells and incubated overnight before the treatment. The cells were treated with the IC50 concentration of dolasetron and ketoprofen and incubated for 48 h. After washing the control and drug-treated wells with PBS, the cells were stained with a mixture of acridine orange and ethidium bromide (100 µg/mL each). The stained cells were visualized and imaged immediately using an inverted fluorescent microscope, and images were captured using the Thermo Fisher scientific EVOS Floid imaging system [25]. For apoptosis, caspase-3/7activity was measured using the Apo-one Homogeneous Caspase-3/7 assay kit (Promega Madison, WI, USA; cat no. G7790), and the manufacturer’s protocol was followed. Cleavage of the non-fluorescent substrate, Z-DEVD-Rhodamine-110, by caspase 3/7 resulted in fluorescent rhodamine-110. The fluorescence of the sample was measured at 530 nm emission and 490 nm excitation in the multimode reader, BioTek [26,27].

### 2.13. Immunofluorescence

HCT116 cells were plated on a coverslip held in a 6-well tissue culture plate, and the cells were allowed to grow overnight. Subsequently, the cells were treated with the IC50 concentration of dolasetron and ketoprofen and incubated for 24 h. Following treatment, the cells were fixed with 10% formalin and permeabilized with 1% Triton X-100 in PBS. Later, the cells were incubated with 5% bovine serum albumin in PBS for 30 min. To detect specific proteins, the cells were incubated with a 1:50 dilution of PUM1 antibody (Abcam, Cambridge, UK; ab92545), DCLK1 antibody (Abcam, Cambridge, UK; cat no. ab37994), and CD133 antibody (Cell Signaling Technology Danvers, Beverly, MA, USA; cat no. 5860s). The primary antibody was detected using a Goat anti-Rabbit IgG (H + L) Cross-Absorbed Secondary Antibody, Alexa Fluor 633 (Thermo Fisher Scientific, Eugene, OR, USA; A21070). Fluorescence images were captured using the EVOS FLoid Imaging System, from Thermo Fisher Scientific [28].

### 2.14. Statistical Analysis

All experiments were performed in triplicate, and the data were statistically analyzed using an unpaired Student’s *t*-test. The results are presented as the mean ± standard error of the mean (SEM). Statistical significance was determined at * *p* < 0.05, ** *p* < 0.01, *** *p* < 0.001, and ^#^ *p* < 0.0001.

## 3. Results and Discussion

### 3.1. Identification of Top Ten Compounds from FDA-Approved Compounds Using Molecular Docking

**Figure 2:**
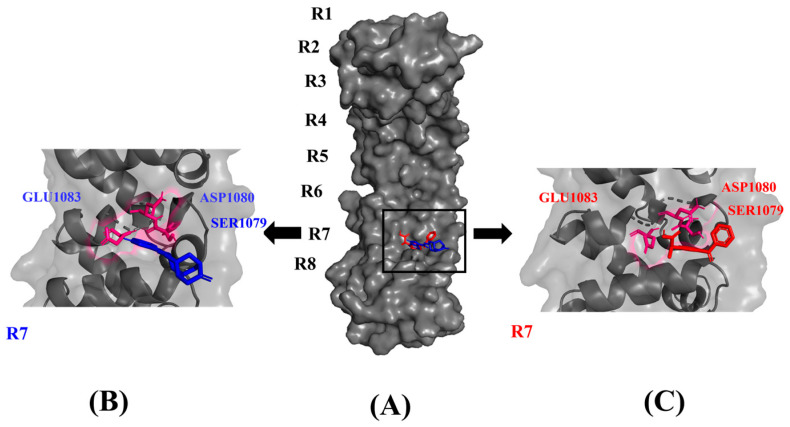
Binding pose of top two FDA-approved compounds (**B**) dolasetron (blue) and (**C**) ketoprofen (red) in the (**A**) RNA binding site of PUM1 protein. Residues Ser1079, Asp1080, and Glu1083 of PUM1 protein that interacts with dolasetron and ketoprofen in the seventh binding site are highlighted in hot pink.

The drug compounds were docked into the proposed binding sites of PUM1 using Autodock Vina and ranked based on their binding affinity. Compounds with lower binding affinity were considered better candidates. We selected the top ten compounds that showed superior binding affinity and were likely to inhibit the interaction of PUM1 with target mRNAs. The binding affinity scores, and hydrophobic interactions of the selected compounds are tabulated in Table 1. Although methsuximide, menadione, and lansoprazole also exhibited favorable binding affinity, they did not form interactions with the proposed binding site residues. The next compound is lefamulin, a semi-synthetic pleuromutilin with a chemical structure that contains a tricyclic core of five-, six-, and eight-membered rings and a 2-(4-amino-2-hydroxycyclohexyl)sulfanylacetate side chain extending from C14 of the tricyclic core. Since it inhibits bacterial protein synthesis by binding to the 50S bacterial ribosomal subunit in the peptidyl transferase center (PTC), this property of Lefamulin makes it a non-suitable candidate as we are targeting colon cancer where a healthy microbiome is needed for a healthy life. Other hits such as allopurinol, ketamine, levocarnitine, and miltefosine have higher binding energy. Among the top ten compounds, dolasetron and ketoprofen demonstrated the highest binding affinity scores compared to the others, along with interactions with the active site residues Ser1079, Asp1080, and Glu1083 of PUM1. This indicates a strong interaction between the ligands and the receptor. The docked poses and interactions of dolasetron and ketoprofen in the PUM1 protein are depicted in Figure 2.

### 3.2. Conceptual Density Functional Theory (C-DFT) Calculations Show Dolasetron and Ketoprofen to Have Better Chemical Reactivity among the Top Ten Compounds

The DFT/B3LYP method with a 6–31 G (2d.p) basis set implemented by the Gaussian method was used to optimize the FDA-approved compounds. Table 2 presents the calculated molecular descriptors of selected FDA compounds, including electronegativity, chemical hardness, softness, chemical potential, and electrophilicity index. A significant characteristic of a compound is a smaller energy gap (ΔE), indicating an easier transition from HOMO to LUMO states. Additionally, a decrease in electronegativity enhances the inhibitory efficiency of compounds. The inhibitory efficiency of a compound depends on factors such as dipole moment, lower electronegativity, and smaller energy gap value. Among the selected FDA-approved compounds, dolasetron and ketoprofen exhibit higher inhibitory efficiency due to their smaller energy gaps (4.06 and 4.93, respectively) and lower electronegativity compared to others. The electron density maps of HOMO and LUMO of the top FDA-approved compounds against PUM1 are shown in Table 2.

### 3.3. Dolasetron and Ketoprofen Are Stable during a 100 ns Simulation Run with Active Interactions with PUM1

**Figure 3:**
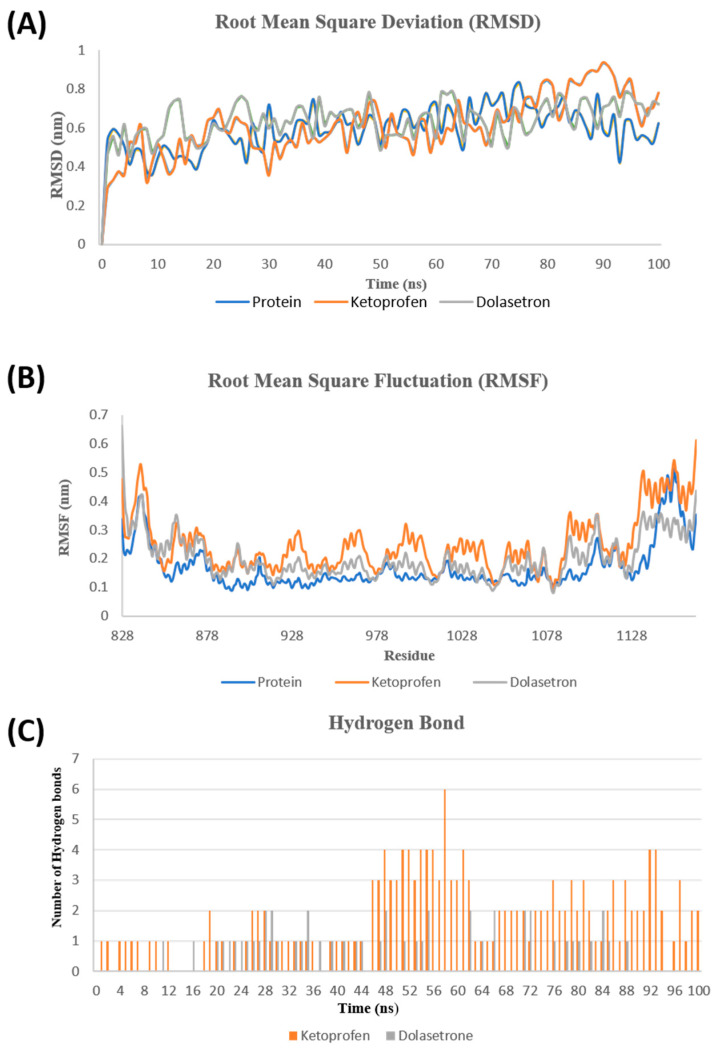
Molecular dynamic simulation graph shows the interaction of protein–ligand molecule over a period of 100 ns. (**A**) Change in the RMSD backbone of the Cα atom of the protein–ligand complex. (**B**) RMSF graph showing the change of PUM1 protein residues. (**C**) Hydrogen bonds formed between protein–ligand complexes.

Molecular dynamics simulations were conducted for 100 ns to study the conformational changes of the protein in the presence of ligands. The stability of the protein was evaluated using RMSD, RMSF, and Hydrogen Bond analysis. The RMSD plot was utilized to assess the stability and conformational changes of the protein over the course of 100 ns. It was calculated for both the drugs and their Apo form as a reference. The RMSD plot indicates that the PUM1 protein underwent minimal conformational changes during the simulation of both complexes. Over the 100 ns period, the drugs remained stable and reached equilibrium. The RMSD of both drugs ranged from 0.2 to 0.6 nm, indicating similar stability. The RMSF fluctuations of each residue were analyzed to determine the effect of the ligand molecules on protein residues during the 100 ns simulation. Residues 1079Ser, 1080Asp, and 1083Glu exhibited higher fluctuations within the protein’s binding site, while other sites in the protein showed minor fluctuations. The number of hydrogen bonds formed between the protein and the drugs was identified and plotted as shown in Figure 3. The residues predicted as binding sites formed hydrogen bonds with the protein throughout the 100 ns simulations.

### 3.4. Dolasetron and Ketoprofen Inhibit Cell Viability, Colony Formation, and Colonospheroids in Colon Cancer Cells

**Figure 4:**
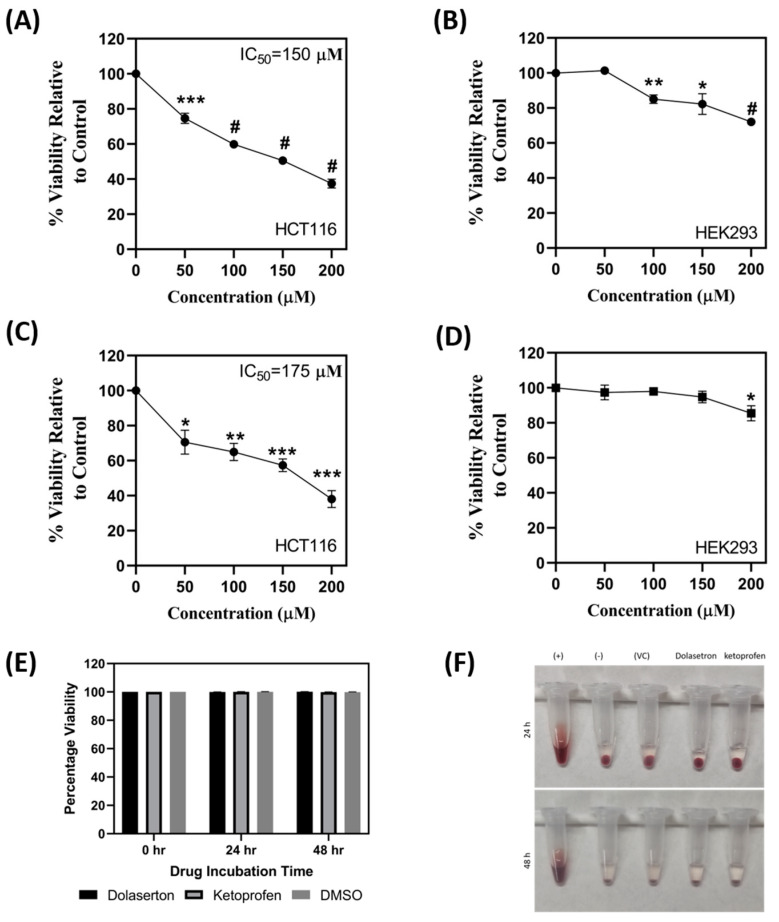
MTT assay and Hemolysis—(**A**,**C**) Viability is reduced when HCT116 colon cancer cells were treated with dolasetron and ketoprofen, respectively. IC50 Value for the compounds was calculated and found to be 150 µM and 175 µM for dolasetron and ketoprofen, respectively. (**B**) and (**D**) HEK293 cells were treated with dolasetron and ketoprofen, respectively. (**E**) There was no hemolysis seen in the treatment group with IC50 concentrations of dolasetron and ketoprofen, distilled water (+), and PBS (−) being used as positive and negative control, respectively. (**F**) Representative image of hemolysis at different time points with positive, negative, and vehicle control. The graph shows mean ± SEM, * *p* < 0.05, ** *p* < 0.01, *** *p* < 0.001, ^#^ *p* < 0.0001.

**Figure 5:**
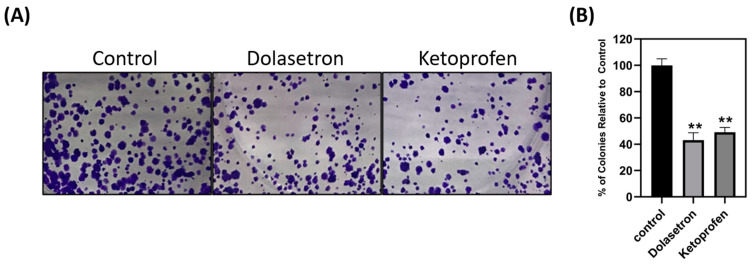
Colony formation assay. (**A**) Wells treated with dolasetron and ketoprofen showed reduced colonies when compared with control wells. (**B**) Graphical representation of the effect of dolasetron and ketoprofen on the percentage of colonies relative to control; graph shows mean ± SEM, ** *p* < 0.01.

**Figure 6:**
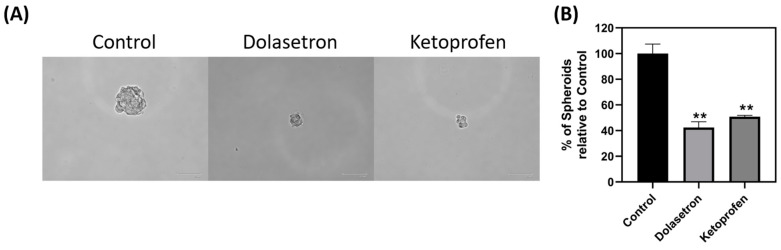
Colonospheroid assay. (**A**) HCT116 cells treated with dolasetron and ketoprofen have shown reduced colonosphere growth when compared with the control. (**B**) Graphical representation of the effect of dolasetron and ketoprofen on the percentage of colonospheroids; graph shows mean ± SEM, ** *p* < 0.01.

To validate the efficacy of the top two FDA-approved compounds in in vitro, dolasetron and ketoprofen were chosen for experimentation on the HCT116 colon cancer cell line. Stock solutions of dolasetron and ketoprofen were prepared in DMSO and used for cell cytotoxicity assays at four different concentrations (50, 100, 150, and 200 µM). The IC50 concentration was determined and utilized in subsequent experiments. MTT assays were performed, and the percentage viability relative to the control was calculated for each drug concentration. The IC50 concentrations of dolasetron and ketoprofen were found to be 150 and 175 µM, respectively, as shown in Figure 4A,C. The potential toxicity of these two compounds with the reported IC50 concentrations was tested using a MTT assay on non-cancerous HEK293 cell line and hemolysis assay. Dolasetron and ketoprofen showed less toxicity in HEK293 cells when compared with HCT116 cells at the same IC50 value. The percentage viability of HEK with dolasetron and ketoprofen treatment is 82.24% and 90% as shown in Figure 4B,D. The percentage viability at IC50 concentrations of both compounds was used to measure the selectivity index (SI). We found that the SI for dolasetron is 1.64, and for ketoprofen it is 1.8 (>1), which shows the selectivity of the drugs to target cancer cells more than non-cancerous cells [29]. We found that the reported IC50 concentration of the drug does not show any toxicity against red blood cells at 24 h and 48 h time points, as shown in Figure 4E,F. This also shows the colony formation assay was conducted to assess the long-term effects of the drugs on colon cancer cells. Figure 5 demonstrates that the number of colonies formed in treated wells was significantly lower than in non-treated wells. Colonospheroid formation, a characteristic of cancer stem cells, was also evaluated. Dolasetron and ketoprofen exhibited a significant reduction in the number and size of colonospheroids in treated wells compared to control wells, as shown in Figure 6.

### 3.5. Dolasetron and Ketoprofen Induce Apoptosis and Reduce PUM1 Expression In Vitro

**Figure 7:**
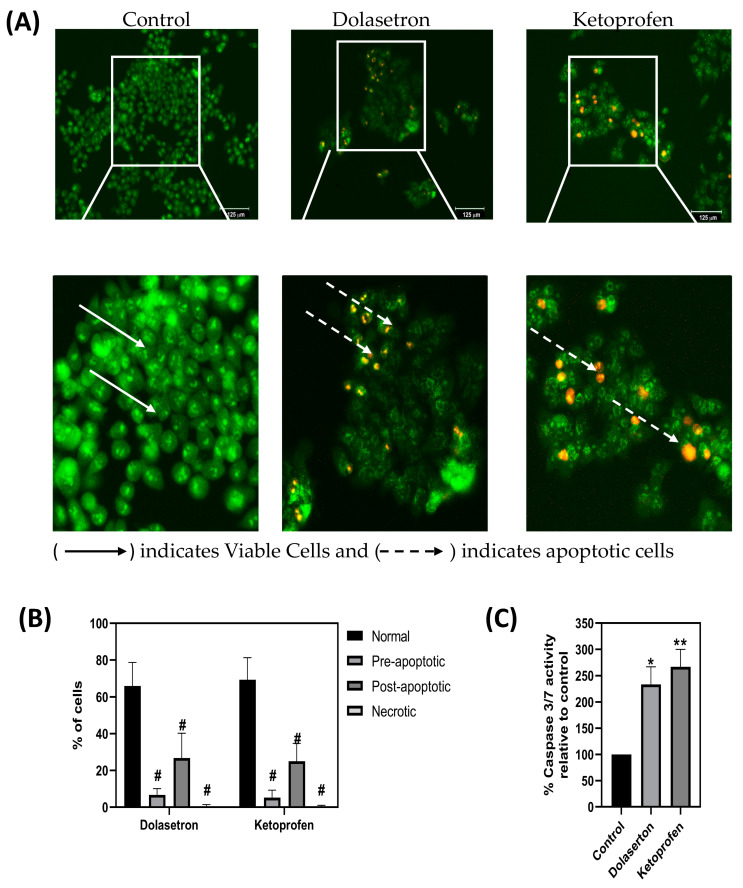
Dual AO/EB fluorescent staining and Caspase 3/7 assay—(**A**) HCT116 cells treated with dolasetron and ketoprofen, the nucleus showed yellow-green fluorescence by acridine orange and orange fluorescence by ethidium bromide staining. The apoptotic cells are observed as orange-red fluorescence. The fluorescence image confirms cells undergo apoptosis once treated with dolasetron and ketoprofen and stained with AO/EB. (**B**) The percentage of cells in normal, pre-apoptotic, post-apoptotic, and necrotic stages were counted from 12 randomized fields of view under a fluorescent microscope. (**C**) HCT116 cells were treated with IC50 concentrations of dolasetron and ketoprofen for 48 h and tested for caspase 3/7 activity. Both compounds induce apoptosis in HCT116 cells when compared to controls. The graph shows mean ±SEM, * *p* < 0.05, ** *p* < 0.01, and ^#^ *p* < 0.0001.

HCT116 cells were subjected to staining with AO/EB post-treatment with dolasetron and ketoprofen. The apoptotic morphology of the stained cells was subsequently examined employing a fluorescent microscope. Notably, the wells that underwent treatment displayed the presence of numerous apoptotic cells, as evidenced by the manifestation of orange-red fluorescence. Conversely, the control wells did not exhibit any cells demonstrating an orange-red hue, thus indicating the presence of viable cells, as depicted in Figure 7A. We have analyzed 12 randomized fields of view under a fluorescent microscope, to count cells in normal, pre-apoptotic, post-apoptotic, and necrotic stages. Figure 7B shows a significant percentage of cells in pre-apoptotic, post-apoptotic, and necrotic stages over the treatment with drugs. Caspase 3/7 plays a vital role as an effector molecule within the apoptosis pathway, triggering a cascade of events that ultimately result in the characteristic features of apoptosis, such as DNA fragmentation and alterations in cellular morphology [30,31]. Dolasetron and ketoprofen have triggered activation of executioner caspase 3/7 in HCT116 cells when compared with untreated controls, as shown in Figure 7C. This suggests dolasetron and ketoprofen induce cell death via a caspase-dependent pathway.

**Figure 8:**
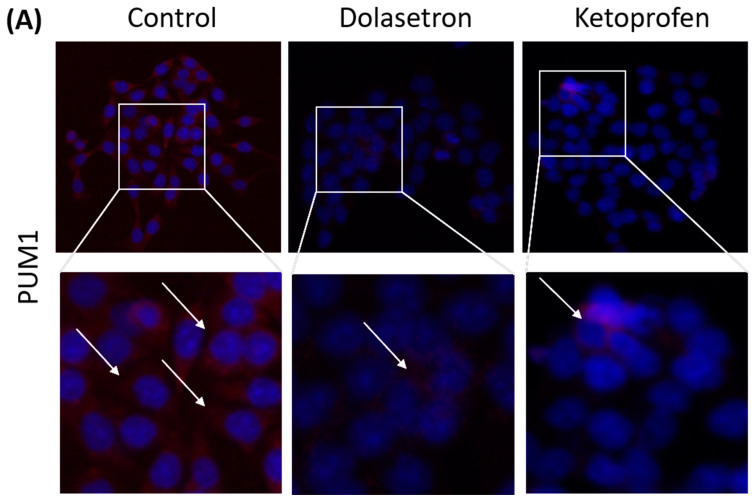

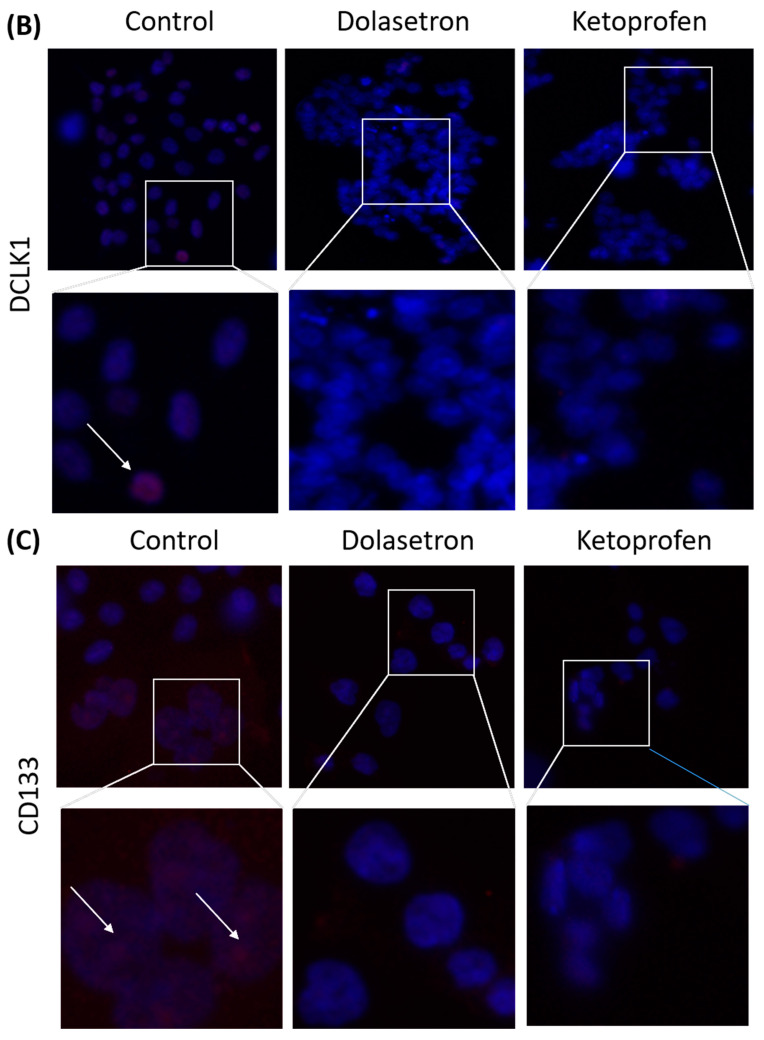
Immunofluorescence assay: HCT116 cells treated with IC50 concentration of dolasetron and ketoprofen for 24 h and used for immunofluorescence. (**A**) The drug-treated group shows reduced PUM1 staining when compared to untreated controls. (**B**,**C**) A similar trend is seen in cancer stem cell markers, reduced staining of DCLK1 and CD133 in the drug-treated group when compared to the untreated control group, respectively. The arrow (→) indicates the expression of the gene in colon cancer cells.

Considering the established involvement of PUM1 in the growth of colon cancer stem cells, we proceeded to investigate the impact of PUM1 inhibition through the administration of dolasetron and ketoprofen. Furthermore, we assessed the effects of these compounds on additional colon cancer stem cell markers, namely DCLK1 and CD133. Our findings revealed a notable reduction in PUM1 expression within the treatment group when compared to the control group. Moreover, the expression levels of the already established cancer stem cell markers were also diminished within the drug treatment group, relative to the control group, as presented in Figure 8. These results signify the inhibitory influence of dolasetron and ketoprofen on colon cancer stem cells in an in vitro setting by targeting PUM1.

## 4. Conclusions

Compounds approved by the Food and Drug Administration (FDA) have undergone extensive safety and toxicity assessments in both human and animal subjects. The prominent benefit of drug repurposing lies in its ability to shorten the duration of drug development and expedite the availability of treatments for patients. The repurposing of dolasetron and ketoprofen as potential therapeutic agents targeting PUM1 represents a promising avenue for intervention in colon cancer treatment. Originally developed as an anti-emetic, dolasetron has demonstrated inhibitory effects on PUM1, while ketoprofen, a nonsteroidal anti-inflammatory drug (NSAID), has shown significant potential in this regard as well. By repurposing these readily available drugs, we can leverage their established anti-inflammatory and anti-emetic properties while concurrently targeting PUM1. Both compounds have exhibited the best binding affinity within the active site of PUM1, accompanied by greater chemical reactivity, smaller energy gaps, and lower electronegativity, as supported by C-DFT calculations. The stability of the protein–ligand complexes were maintained throughout the 100 ns molecular dynamics simulation, while dolasetron and ketoprofen displayed notable inhibitory effects on the viability of colon cancer cells and colony formation in vitro. These compounds have shown a cytotoxicity effect in HCT116 cells and lower cytotoxicity in HEK293 cells with SI > 1 showing more selectivity against targeting cancerous cells. These compounds do have long-term effects on the cancer cells as the number of colonies -were reduced significantly when compared with the untreated control group. In the colonospheroid assay where colon cancer stem cells are enriched, these drugs have shown their potential to reduce the number and size of colonospheroids significantly. Identified compounds also have a potential to induce apoptosis via activating the executioner caspase 3/7 significantly when compared with the untreated control. Drug treatment also reduced PUM1 expression and cancer stem cell marker (DCLK1 and CD133) expression in colon cancer cells.

Our findings show the potential of dolasetron and ketoprofen in inducing cytotoxicity in colon cancer and cancer stem cells by targeting PUM1. Dolasetron and ketoprofen show promise as therapeutic agents for the treatment of colon cancer and colon cancer stem cells by apoptotic cell death via activating the caspase-dependent pathway. Given that dolasetron is already utilized as a supportive therapy in chemotherapy to mitigate side effects, its incorporation into current treatment regimens may be justified due to its anticancer and anticancer stem cell properties in the context of colon cancer [32]. However, our immunofluorescence experiment shows a reduction in protein staining of treated groups. Further Western blot analysis is required at understanding the direct expression of PUM1 and its target gene after treatment with these drugs. More research and clinical trials are imperative to evaluate the efficacy and safety of these drugs in these specific contexts.

## Figures and Tables

**Table 1:** Binding affinities of the top FDA-approved compounds on the PUM1 protein with interacting amino acid residues contributing towards hydrophobic interactions. The top two compounds have been highlighted (bold).

Sr. No.	Compound Name	Binding Affinity (Kcal/mol)	Hydrophobic Interactions
**1**	**Ketoprofen**	**−8.2**	Val894, Val896, Leu1014, Tyr1120, Thr1021, Tyr1123, Leu1026, Ile1046, Ser1079.
**2**	**Dolasetron**	**−7.4**	
3	Methsuximide	−6.3	
4	Menadione	−6.3	
5	Lansoprazole	−6.3	
6	Lefamulin	−6.0	
7	Allopurinol	−5.3	
8	Ketamine	−5.2	
9	Levocarnitine	−4.2	
10	Miltedosine	−3.4	

**Table 2:** Statistics of the conceptual DFT-global reactivity descriptors and their derivatives of the best FDA-approved compounds against PUM1. Electron density maps of HOMO and LUMO of top FDA-approved compounds against PUM1. Some abbreviations for the table; HOMO/LUMO energy gap = ΔE, Absolute Hardness = η, Global Softness = σ, Electronegativity = χ, Chemical potential = μ, Electrophilicity index = ω.

Sr. No.	Compound Name	Total Energy (In eV)	Molecular Dipole Moment (Debye)	E_HOMO_	E_LUMO_	ΔE	η	σ	χ	μ	ω	HOMO	LUMO
1	Dolasetron	−22960	2.66	−6.6	−1.6	4.9	2.5	0.2	−4.1	4.1	3.38	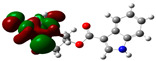	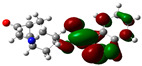
2	Ketoprofen	−29149	5.05	−5.6	−1.5	4.1	2	0.2	−3.6	3.6	3.13	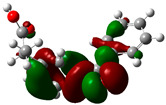	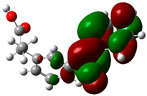
3	Methsuxmide	−18241	0.73	−6.5	−1	5.6	2.8	0.2	−3.8	3.8	2.52	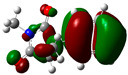	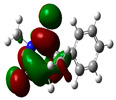
4	Menadione	−15567	5.3	−5.8	−3.7	2.2	1.1	0.5	−4.7	4.7	10.5	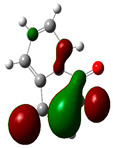	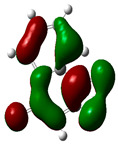
5	Lansoprazole	−44366	2.83	−5.5	−2	3.4	1.7	0.3	−3.7	3.7	4.06	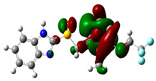	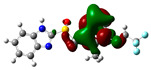
6	Lefamulin	−52329	3.77	−6	−0.9	5.1	2.5	0.2	−3.5	3.5	2.38	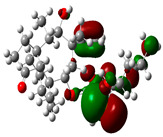	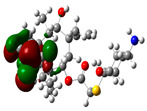
7	Allopurinol	−13256	3.69	−6.8	−1.3	5.5	2.8	0.2	−4.1	4.1	3.02	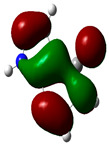	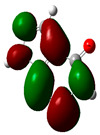
8	Ketamine	−29219	9.37	−1.9	−1	0.9	0.5	1.1	−1.4	1.4	2.17	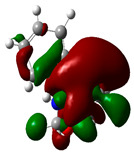	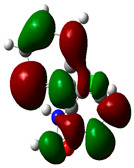
9	Levocarnitine	−15128	20.44	−2.6	−0.2	2.4	1.2	0.4	−1.4	1.4	0.82	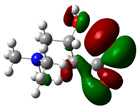	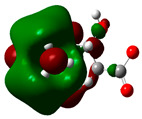
10	Miltefosine	−41496	19.23	−3.7	−0.1	3.6	1.8	0.3	−1.9	1.9	1.02	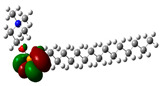	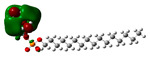

## Data Availability

Data will be made available on request.
